# A Web-Based Platform to Collect Data from ESRD Patients Undergoing Dialysis: Methods and Preliminary Results from the Brazilian Dialysis Registry

**DOI:** 10.1155/2018/9894754

**Published:** 2018-03-05

**Authors:** Jocemir R. Lugon, Pedro A. Gordan, Fernando S. Thomé, Antonio A. Lopes, Yoshimi J. A. Watanabe, Carmen Tzanno, Ricardo C. Sesso

**Affiliations:** ^1^Universidade Federal Fluminense, Niterói, RJ, Brazil; ^2^Universidade Estadual de Londrina, Londrina, PR, Brazil; ^3^Universidade Federal do Rio Grande do Sul, Porto Alegre, RS, Brazil; ^4^Universidade Federal da Bahia, Salvador, BA, Brazil; ^5^Hospital São João de Deus, Divinópolis, MG, Brazil; ^6^Sociedade Brasileira de Nefrologia, São Paulo, SP, Brazil; ^7^Universidade Federal de São Paulo, São Paulo, SP, Brazil

## Abstract

**Introduction:**

The methods and initial results of a web-based platform to collect data from patients receiving maintenance dialysis in Brazil are reported.

**Methods:**

Companies providing management software for dialysis centers adapted their system to comply with a formulary of the Brazilian Society of Nephrology. Baseline and follow-up individual patients' data were transmitted via Internet on monthly bases to the coordinating center from 2011 to 2017.

**Results:**

73 dialysis centers provided information of 24,930 patients: 57% were male, 28% were 64 years old or older, and 13% were overweight/obese. Median dialysis vintage was 28 months. Hemodialysis was the most frequent initial therapy (93%) with venous catheters used in 64% of cases. Conventional hemodialysis remained the main current therapy (90%). Seropositivity for hepatitis C, hepatitis B, and HIV was 2.7%, 1.1%, and 0.5%, respectively. Erythropoietin (53.9%), iron (35.1%), and sevelamer (23.4%) were the most used medications. Hemoglobin < 100 g/L and serum P > 1.74 mmol/L were present in 33.1% and 36.6% of the cases, respectively. The 5-year survival of incident cases (*n* = 7,538) was 57%.

**Conclusion:**

The initiative represents an innovative strategy to collect clinical and epidemiologic data of dialysis patients which may be applied to other settings and provides information that can contribute to guiding clinical practice and health care policy.

## 1. Introduction

The incidence and prevalence of end-stage renal disease (ESRD) requiring renal replacement therapy (RRT) either by dialysis or transplant have increased progressively reaching “epidemic proportions” in the world [1–4]. The high cost of treating these patients represents a major concern for the public health systems, even in developed countries [5–7]. In Brazil, for example, the government spends about 4% of the budget of the Ministry of Health in the treatment of patients undergoing RRT who represent less than 0.1% of the population.

Most of the information available regarding the epidemiology of ESRD in the world is derived from developed countries. Geographical variations of treatment rates and cultural and financial aspects related to access of RRT are important features that are not properly addressed in the literature. The Brazilian Society of Nephrology (BSN) understands that local information regarding the epidemiology of ESRD is crucial to improve both public health policies regarding chronic kidney disease and clinical practice by general practitioners and specialists.

The BSN has been obtaining information regarding dialysis treatment practices in Brazil since 1999, adopting the dialysis centers as the working unit [8, 9] using a strategy of collecting once a year grouped data per dialysis center in a simple questionnaire containing basic patients' data. For decades it has been considered of interest to the BSN to develop a project in which the aim was to have individual patient information, encompassing a greater number of epidemiological, clinical, and laboratory characteristics, and follow-up data on outcomes. Initiatives of partnerships with the Ministry of Health of Brazil in this direction have not been successful so far. As most of the dialysis centers in Brazil use computerized data collection based on systems developed by software companies, it was thought that a collaboration involving these companies and the BSN could contribute to achieving the objective of a successful Internet-based platform to collect and analyze individual data of ESRD patients undergoing chronic dialysis.

We describe here the methods to develop and implement the Brazilian Dialysis Registry (BDR). In addition, the preliminary results regarding patient's characteristics, treatment, and survival are reported.

## 2. Methods

In 2010, the working committee of BSN for the Brazilian Dialysis Registry developed an electronic database to collect clinical and epidemiological information from ESRD patients undergoing dialysis therapy in the country. The primary objective was to create a national database receiving standardized patients' information (baseline and follow-up) directly from dialysis centers through the Internet, with minimal workload from the personnel at the dialysis units. For this purpose, a database server was set at the BSN secretary in São Paulo, where a coordinating center of the registry was located.

Initially we contacted most of the largest companies that provided software for patients' management at dialysis centers all over the country. Several of the companies agreed to participate in this initiative and adapted their software system to conform to our standard registry framework developed by the study coordinating committee. The data framework consisted of a minimum of selected patients' features, most of them routinely collected for the electronic patient records. In addition, the systems of each software company were adjusted to electronically transmit patients' information to the coordinating center via Internet. The standard patients' information in the database contained sociodemographic data, clinical characteristics, primary renal diagnosis, features of dialysis therapy, comorbid conditions, routine laboratory tests, medications, and last patients follow-up information and date concerning being alive on therapy, dead, or having received a renal transplant.

The participating companies were the following: Nefrodata©, Hemosys©, Nefrosoft©, Nefrosys©, Dialsist©, and Fresenius©. There was an initial period of tests for the companies to make the needed adjustments in their system and verify, along with the supervision of a technician in informatics of the BSN registry, the feasibility and reliability of the data transmission. The dialysis centers using a tested software system from any of these companies were eligible to be enrolled in the registry. After receiving a tutorial with the needed information for the procedures, the professional in charge in each dialysis center was able to send patients' information to the registry database via Internet. The initial performance of the procedures, the difficulties in sending the data files to the coordinating center, errors regarding specific questions or invalid answers, missing data, and the reliability of the process were checked and rechecked with an information technology technician supervising the system at the registry center. In particular, continuous data (e.g., age and laboratory parameters) out of acceptable range and excess of missing data in the files were checked by the information technology technician who, whenever needed, contacted the person responsible for the center to solve these and other possible questions related to the files received. The* modus operandi* of the system requires monthly updates of patients' information from every participating center by the professional in charge. This procedure was usually not time-consuming and was easily done by the centers. For the purpose of the registry, the person responsible for the center did not have to digitize any additional information; he just had to send what was already stored in the electronic patient records. Every 2-3 months the participating centers received an e-mail from the coordinating center at the BSN reminding them to upload the last information if they had not done so.

Since 2011, among all the 727 chronic dialysis centers in the country registered in the BSN files, 145 were randomly selected stratified by geographic region and invited to participate in the registry. As the response rate of these initially selected centers was low, the coordinating committee decided to accept the inclusion of any center among all the 727 centers whose responsible manager demonstrated willingness to participate, as long as one of the tested software systems was used for the data collection and technical requirements were met. In addition, the BSN started to announce through their media (website, journals, and meetings) the existence of the initiative and its objectives, encouraging the directors of dialysis centers to participate in the project.

For the present report, data collected from January 2011 through July 2017 were included. The Institutional Review Board of the Federal University of São Paulo approved the study. The responsible managers of the participating centers gave their written agreement to participate in the registry. As the initial information of a patient reached the database center, data were deidentified; he/she received a unique identification number, precluding patient's name identification. Confidentiality about all information of each patient in the registry was guaranteed. No one had access to the database, except the working committee of BSN for the Brazilian Dialysis Registry. The funding agency did not have access to the database.

Data are reported in a descriptive form. Survival curves using the Kaplan-Meier method were calculated for the whole group of incident cases and stratified by gender. The starting point of follow-up was the date of chronic dialysis initiation and the end was the date of death, kidney transplantation, or the last follow-up, whichever came first. Survival curves were compared by the log-rank test.

## 3. Results

From the 145 randomly selected dialysis centers invited, 33 agreed to participate. Forty additional centers participated voluntarily, resulting in an overall number of participating centers of 73. They correspond to 10.1% of the total number of dialysis centers of the country (*n* = 727) and about the same proportion of the overall patients available in the period. The regional distribution of these 73 centers was close to the proportion of all active centers per region in the country, although there was an overrepresentation of the South and no participation of the North (Table 1).

Overall, 24,930 patients were included in the database and presented in this report. 70% of the patients (*n* = 17,392) had begun chronic dialysis prior to the inclusion in the registry (prevalent cases) and 30% (*n* = 7,538) were new cases (incident cases). Median (IQR) time on dialysis was 28 (9–64) months.

The response rate for the several patients' characteristics presented varied widely in the database. For most features, the response rate was above 75%, except for employment situation (36%), modality of the first dialysis program (35%), initial vascular access at hemodialysis start (28%), cardiovascular comorbidities (58%), and laboratory parameters (66%). For the current dialysis modality, the percentage of response was 88%. As the response rates for employment situation, initial dialysis modality, and access at dialysis start were low, these results should be interpreted with caution. Data for the whole group of patients that are shown in the tables represent percentages of the available answers.

The majority of the patients were on chronic dialysis programs funded by the Government; most were male, had white skin color, and retired due to the illness. Patients' age (mean ± SD) was 54.3 ± 16.7 years. 69% of the patients were between 20 and 64 years, only 2.2% were below 20 years, and those who were 65 years old or older accounted for 29.1% of the sample. The body mass index at dialysis start indicated that 9.3% of the patients were malnourished, 27% were overweight, 12.2% were obese, and 1.0% were morbidly obese (Table 2).

The great majority of patients underwent hemodialysis as the initial renal replacement therapy modality (94.3%), and their main vascular access was a central venous catheter (64%); only 35.2% used a native arteriovenous fistula. As for the current dialysis modality, conventional hemodialysis continued to be used by most patients (90%), and “more frequent” hemodialysis was used by 3.6% of the patients. The majority of the patients in this sample were on dialysis therapy for one to five years, and 9.3% were on treatment for more than 10 years (Table 3).

The most commonly reported primary renal disease was hypertensive nephropathy (18.6%) followed by diabetic kidney disease (16.8%) and chronic glomerulonephritis (7.4%). Overall, the report rates of comorbidities by the centers were low. Besides hypertension, diabetes and cardiac diseases were the most prevalent ones (11.7% and 7.3%, resp.). The percentages of patients who tested positive for hepatitis C, hepatitis B, and HIV virus were 2.7%, 1.1%, and 0.5%, respectively. Among the selected medications used by the patients, erythropoietin (53.9%), iron IV (35.1%), sevelamer hydrochloride (23.4%), and acetylsalicylic acid (19.3%) were the most frequent ones. Calcium carbonate/acetate and statins were used by 17.8% and 14.4% of the patients, respectively (Table 4).

Among the most recently available laboratory parameters, hemoglobin levels were <100 g/L or >130 g/L in 33.1% and 10.8% of the patients, respectively, serum calcium was <2.13 mmol/L or >2.60 mmol/L in 24.2% and 7.1%, respectively, serum phosphorus was <1.13 mmol/L or >1.74 mmol/L in 13.9% and 36.6%, respectively, PTH was <150 ng/L or >600 ng/L in 29.3% and 30.7%, respectively, and the urea reduction rate was <65% in 38.5% of the patients (Table 5).

**Figure 1:**
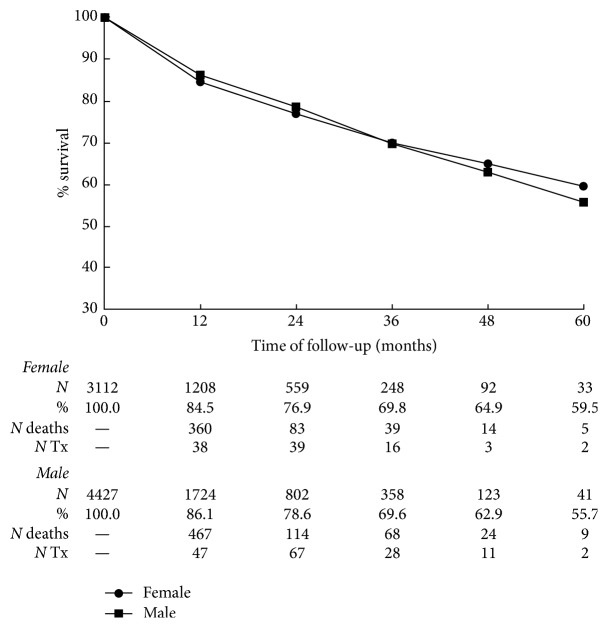
Survival curves for incident dialysis patients by gender. The number of patients at risk in the beginning of each time interval and the percent survival are given at the bottom of the graph as well as the number of deaths and kidney transplants after each period by gender. *P* = 0.571 for the comparison between the curves.

Figure 1 shows survival curves for the incident cases (*n* = 7538, 58.3 ± 16.3 years of age, 21.4% diabetics). The overall percentage of survival was 85% at one year and 56.7% at 5 years of follow-up, leaving us with a mean annual mortality rate for the analyzed period close to 8%. The survival curves stratified by gender (no significant difference in mean age or percentage of diabetics was present between genders) overlap until 3.5 years of follow-up and thereafter women tended to have slightly better five-year survival than men but statistical significance was not found (59.5% versus 55.7%, resp., *P* = 0.571). During the study period, there were 684 deaths and 158 patients received a transplant among men; the corresponding numbers for women were 501 and 98, respectively.

## 4. Discussion

Information as to the epidemiology of ESRD in the country may be of utmost importance to the development of strategies to address this relevant public health problem. This is the first report of the Brazilian Dialysis Registry, a web-based platform to collect individual data from dialysis patients treated in all regions of the country. Initiated in 2011, the registry collected data of approximately 25,000 patients undergoing regular dialysis in Brazil. The results generated by the data from this national registry should be viewed as important pieces of information to guide clinical practices and development of policies to address one of the most relevant public health problems, that is, the increasing prevalence of ESRD requiring maintenance dialysis treatment [10].

The setup of the system, in which the data is transmitted electronically to the BSN and anonymously and confidentially stored in the database center, was only possible due to a successful cooperation between researchers and technicians of information technology working for either the BSN or management software companies. The strategy that, as far as we could know, is until now unique may be especially suited for a large dimension country such as ours, in which personal contact with every dialysis center can be troublesome. However, we think that its application to small countries can be equally easier.

Despite our efforts, we received answers from only 23% of the invited centers. We wonder if the low response rate was related to the lack of familiarity with the technology and/or to the fact that a specific person is required to assume the responsibility for sending the information. Efforts have been undertaken to better understand and remedy these difficulties. It is our expectation to see an increased participation rate in the future as the nephrology community realizes how simple and quick is the procedure.

It should be acknowledged that although the database comprises information collected from the different regions of the country, by the time the present report was generated, the South was overrepresented and centers from the North were not engaged in the initiative. The North region is the one with the lower number of dialysis centers in the country (*n* = 37), corresponding to only 5% of the total. These drawbacks do not detract from the data as a whole, and the almost 25,000 patients in the database represent a large sample, which allows a valuable preliminary overview of the ESRD patients on chronic dialysis in Brazil. The response rates for a number of specific topics were low and worrisome. It is our view that such obstacle can be surpassed in the future by asseverating a continuous feedback to the centers engaged in the project.

Consistent with most of the epidemiological data derived from either Brazil [10] or different countries [11], patients were predominantly male. Most of them were between 45 and 64 years by the time of their entrance in dialysis but an expressive fraction (29%) was over 65 years of age. The proportion of white patients was close to the general Brazilian population [12]. As expected, the vast majority of the treatments (74%) were funded by the public health system. It should be commented though that the fraction of treatments funded by private health care companies (26%) is a little bit lower than the national estimate of owners of private health insurance, which is thought to be around 30% [12]. As previously mentioned, there was an overrepresentation of the South (a relatively high income per capita region) and as a result there was a larger proportion in the sample of patients derived from dialysis companies that privilege entrance of owners of private health insurance.

Confirming the low rate of work capacity rehabilitation of the dialysis treatment, only 15% of patients informed to be actively working, with a substantial proportion of them retired due to the age or disease. However, this question had a low rate of response and the results should be interpreted with caution. In contrast to the historical view in which malnutrition was a major worry among dialysis patients [13], the majority of the studied patients outside the margins of normal BMI were found to be on the high side of the scale being overweight (27%) or obese (13%); undernourishment represented only 9% of the sample. These findings are in agreement with data derived from developed countries [11, 14]. The proportion of overweight and obesity found in the present report highlights the need to address their prevention and treatment to overcome their associated comorbidities. We wonder if a similar scenario may be present in other developing countries nowadays, but information on this respect in the literature is scarce.

Hemodialysis was the major modality (94%) employed for the start of dialysis program and a central intravenous catheter was used as the vascular access in most of the instances (64%). However, caution should be exercised regarding this last number due to the low report rate of this data. In agreement with most of countries in the world [11], conventional hemodialysis continues to be the main modality of the dialysis to treat ESRD patients in Brazil (90%). Interestingly, 9% of patients are undergoing dialysis treatment for 10 or more years, perhaps reflecting an overall good quality of care. In contrast to reports from many developed countries [11], in which diabetic kidney disease is the preponderant primary kidney disease, hypertension remains the lead cause of ESRD in Brazil. Of note, the percentage of undetermined causes remains high and close to 50%, what may have impaired the accuracy of these estimates.

When looking at comorbidities, we should keep in mind that this item was the one with the lowest rate of answering in the report forms limiting the validity of the information due to subnotification. In spite of that, the prevalence rates of positive serology for hepatitis B and hepatitis C are very close to the ones found in the last Brazilian Dialysis Census [10], allowing us to think that, for this specific topic, the obtained numbers may be reliable. It is noteworthy that the high prevalence rates of these viral infections seen in the past [8, 9] decreased consistently in the last 10–15 years and are now approaching the numbers for the general population as a consequence of reduction of the blood transfusion secondary to the erythropoietin availability and adoption of universal environmental precautions and, in case of hepatitis B, to the development of an effective vaccine.

As far as medications are concerned, sevelamer hydrochloride was the preferred phosphate binding agent with a frequency of use which exceeded the one of the calcium-based agents. Calcitriol (especially the oral preparation) far prevailed over the other options to supplement vitamin D. An expressive fraction of patients was receiving aspirin (19%) and/or statins (14%). It should be noted, however, that the effectiveness of initiation of statins to improve outcomes for dialysis patients has not been supported by clinical trials, while aspirin use may be beneficial in the secondary prevention of cardiovascular events in selected patients [15, 16]. Although intravenous iron and erythropoietin are supplied by the Brazilian government, the data of Brazilian Dialysis Registry indicate that 44% of patients did not reach the recommended target range for hemoglobin with the majority of them sitting in the low range (33%). Phosphate levels and PTH levels exceeded 1.74 mmol/L and 600 ng/L in 37% and 31%, respectively, but these results are not unexpected considering that cinacalcet is not provided by our public health system yet.

Finally, for the first time, it was possible to obtain a Kaplan-Meier survival curve of a national sample of incident dialysis patients in Brazil. As a whole, the 5-year survival rate of the patients (57%), especially taking into consideration the fact that the mean age at entrance was 58.5 ± 20.1 years, is notable. The mean annual mortality rate of the analyzed period of time is close to 8% and is comparable to the best reported values all over the world [11, 17, 18]. Women tended to have slightly better survival rate after 4 years but the difference was not statistically significant.

This study has several limitations. The data collection was not randomly obtained, limiting the generalizability of the findings. However, the distribution of the participating centers, except for the lack of those from the North region (the less densely populated and with the lowest number of centers, 5% of the country), is relatively close to the actual distribution of the centers in the country. We continue to dedicate efforts to include more centers in the registry and increase the representativeness of the sample. An administrative secretary of the registry at the BSN using all contact media of the society (website, e-mail, journals, and meetings) has constantly been inviting the dialysis centers' managers to participate in the initiative. As previously mentioned, our intention was to collect the information that was already available in the dialysis centers' database to decrease the workload and time spent with the generation of information for the registry. Unfortunately, even adopting such strategy, the process of sending information monthly to the BSN was not consistently uniform, resulting in the fact that about 15%–20% of participating centers were more than 3 months delayed with this routine.

On the other side, the study has several strengths. The use of a web-based platform for the present purpose has been proven to be feasible. The process was easy to perform, was not time-consuming, was very convenient for a large country such as Brazil, and was appropriate for the longitudinal follow-up of patients. In addition, the strategy allows prompt analysis of the data after their arrival at the central database and guaranteed anonymity and confidentiality. Moreover, this study reports several important clinical-epidemiologic aspects in a very large sample (about 25,000) of patients on dialysis in the country.

## 5. Conclusions

The RBD represents an innovative strategy to collect clinical and epidemiologic data of chronic dialysis patients which may also be applied to other settings. It allowed the collection and analysis of a variety of individual patient parameters, providing pivotal information that can contribute to improvements in both clinical practice and health care policy planning.

## Figures and Tables

**Table 1:** General features of the program.

Year of conception	2006
Data collection dates	Jan/2011–Jul/2017
Number of invited centers	145
Number of participant centers/total (%)	73/726 (10.1)
Volunteers	40
Randomly assigned	33
Regional distribution of centers, *n*/*N* total per region (%)	
North	0/37 (0)
Midwest	6/66 (9.1)
Northeast	12/130 (9.2)
Southeast	32/340 (9.4)
South	23/153 (15.0)
Companies involved^*∗*^ , *n*	6

^*∗*^Nefrodata©, Hemosys©, Nefrosoft©, Nefrosys©, Dialsist©, and Fresenius©.

**Table 2:** General characteristics of patients.

Total number	24,930
Prevalent patients, *n* (%)	17,392 (69.7)
Incident patients, *n* (%)	7,538 (30.3)
Male gender, %	57.2
Funding by the Public Health System, %	74.2
Age at dialysis start, years, %	
6–<12	0.2
12–<20	2.0
20–<45	25.0
45–<65	43.7
65–<75	17.6
≥75–97	11.5
Skin color (white/nonwhite, %)	53.7/46.3
Body mass index at entrance, kg/m^2^, %	
<18.5	9.3
18.5–≤25.0	50.4
>25–≤30	27.0
>30–≤40	12.2
>40	1.0
Employment situation, %	
Full-time job	13.3
Part-time job	2.1
Retired	69.5
Other	15.1

**Table 3:** Selected dialysis features.

Modality of 1st dialysis in life, %	
Hemodialysis	94.3
CAPD	2.4
APD	2.2
IPD	1.1
Vascular access at hemodialysis start, %	
Native fistula	35.2
Graft	0.2
Central venous catheter	64.4
Current dialysis modality, %	
Conventional hemodialysis	90.0
More frequent hemodialysis (≥4 sessions/week)	3.6
CAPD	3.1
APD	3.2
Dialysis vintage, months, %	
≥3–12	18.8
>12–60	43.1
>60–120	17.7
>120–180	6.1
>180	3.2

**Table 4:** Primary renal disease, comorbidities, and medications.

Primary renal disease (*n* = 22,136)	
Hypertensive nephropathy	18.6
Diabetic kidney disease (type 2)	11.7
Diabetic kidney disease (type 1)	5.1
Chronic glomerulonephritis (except SLE)	7.4
Adult polycystic kidney disease	3.3
Chronic interstitial nephritis	2.4
SLE nephropathy	0.7
Myeloma related kidney disease	0.3
Other	28.9
Undetermined	21.7
Comorbidities (*n* = 21,183)^*∗*^	
Arterial hypertension	23.3
Diabetes on insulin	6.4
Diabetes without medication	3.7
Diabetes on oral drugs	1.6
Coronary artery disease	3.4
Congestive heart failure	1.9
Other cardiac diseases	2.0
Stroke	1.2
Limb amputation	0.6
Previous kidney transplantation	1.8
Chronic obstructive pulmonary disease	0.4
Positivity for hepatitis C serology	2.7
Positivity for hepatitis B serology	1.1
Positivity for HIV positivity serology	0.5
Smoking	3.0
Malignancy	0.9
Inability to walk	1.0
Support needs for daily activities	2.3
Medications (*n* = 21,390)	
Erythropoietin	53.9
Intravenous iron	35.1
Calcium carbonate/calcium acetate	17.8
Oral calcitriol	10.1
Intravenous calcitriol	2.9
Sevelamer hydrochloride	23.4
Oral vitamin D, nonactive form	4.9
Cinacalcet	3.7
Statins	14.4
Acetylsalicylic acid	19.3

Parenthesis content denotes the number of available responses for each item.  ^*∗*^For coronary artery disease, *n* = 14,538; for other cardiac diseases, *n* = 14,542.

**Table 5:** Selected laboratory parameters.

Hemoglobin, g/L (%)	
<100	33.1
100–130	56.1
>130	10.8
Ferritin, pmol/mL	1618 (652–4186)^a^
Calcium, mmol/L (%)	
<2.13	24.2
2.13–2.60	68.7
>2.60	7.1
Phosphorus, mmol/L (%)	
<1.13	13.9
1.13–1.74	49.5
>1.74	36.6
Parathyroid hormone, ng/L (%)	
<150	29.3
150–600	40.0
>600	30.7
Creatinine, *µ*mol/L	734 ± 318^b^
Urea reduction rate (%)	
<65%	38.5
≥65%	61.5
Alanine aminotransferase, *µ*kat/L	0.30 (0.17–1.17)^a^
Albumin, g/L	37 ± 6^b^

^a^Median (interquartile range).  ^b^Mean ± standard deviation.
